# Habitual coffee consumption and cognitive function: a Mendelian randomization meta-analysis in up to 415,530 participants

**DOI:** 10.1038/s41598-018-25919-2

**Published:** 2018-05-14

**Authors:** Ang Zhou, Amy E. Taylor, Ville Karhunen, Yiqiang Zhan, Suvi P. Rovio, Jari Lahti, Per Sjögren, Liisa Byberg, Donald M. Lyall, Juha Auvinen, Terho Lehtimäki, Mika Kähönen, Nina Hutri-Kähönen, Mia Maria Perälä, Karl Michaëlsson, Anubha Mahajan, Lars Lind, Chris Power, Johan G. Eriksson, Olli T. Raitakari, Sara Hägg, Nancy L. Pedersen, Juha Veijola, Marjo-Riitta Järvelin, Marcus R. Munafò, Erik Ingelsson, David J. Llewellyn, Elina Hyppönen

**Affiliations:** 1 Australian Centre for Precision Health, University of South Australia, Adelaide, Australia; 2 MRC Integrative Epidemiology Unit (IEU) at the University of Bristol, Bristol, UK; 3 UK Centre for Tobacco and Alcohol Studies (UKCTAS) and School of Experimental Psychology, University of Bristol, Bristol, UK; 4 Center for Life Course Health Research, University of Oulu, Oulu, Finland; 5 Oulu University Hospital, Oulu, Finland; 6 Department of Medical Epidemiology and Biostatistics, Karolinska Institutet, Stockholm, Sweden; 7 Research Centre of Applied and Preventive Cardiovascular Medicine, University of Turku, Turku, Finland; 8 Helsinki Collegium for Advanced Studies, Helsinki, Finland; 9 Department of Psychology and Logopedics, Faculty of medicine, University of Helsinki, Helsinki, Finland; 10 Department of Public Health and Caring Sciences, Clinical Nutrition and Metabolism. Uppsala University, Uppsala, Sweden; 11 Department of Surgical Sciences, Orthopaedics, Uppsala University, Uppsala, Sweden; 12 Institute of Health & Wellbeing, University of Glasgow, Glasgow, UK; 13 Unit of Primary Health Care, Oulu University Hospital, Oulu, Finland; 14 Department of Clinical Chemistry, Fimlab Laboratories and Finnish Cardiovascular Research Center Tampere, Faculty of Medicine and Life Sciences, University of Tampere, Tampere, Finland; 15 Department of Clinical Physiology, Tampere University Hospital and Faculty of Medicine and Life Sciences, University of Tampere, Tampere, Finland; 16 Department of Pediatrics, Tampere University Hospital and Faculty of Medicine and Life Sciences, University of Tampere, Tampere, Finland; 17 Department of Public Health Solutions, National Institute for Health and Welfare, Helsinki, Finland; 18 Wellcome Centre for Human Genetics, Nuffield Department of Medicine, Oxford, OX3 7BN, UK; 19 Department of Medical Sciences, Cardiovascular Epidemiology, Uppsala University, Uppsala, Sweden; 20 Population, Policy and Practice, UCL Great Ormond Street Institute of Child Health, London, WC1N 1EH UK; 21 Department of General Practice and Primary Health Care, University of Helsinki and Helsinki University Hospital, Helsinki, Finland; 22 Folkhälsan Research Center, Helsinki, Finland; 23 Department of Clinical Physiology and Nuclear Medicine, Turku University Hospital, Turku, Finland; 24 Department of Psychiatry, Research Unit of Clinical Neuroscience, University of Oulu, Oulu, Finland; 25 Department of Psychiatry, University Hospital of Oulu, Oulu, Finland; 26 Department of Epidemiology and Biostatistics, MRC–PHE Centre for Environment & Health, School of Public Health, Imperial College London, London, UK; 27 Biocenter Oulu, University of Oulu, Oulu, Finland; 28 Department of Medicine, Division of Cardiovascular Medicine, Stanford University School of Medicine, Stanford, CA 94305 USA; 29 Department of Medical Sciences, Molecular Epidemiology and Science for Life Laboratory, Uppsala University, Uppsala, Sweden; 30 Stanford Cardiovascular Institute, Stanford University, CA, 94305 USA; 31 University of Exeter Medical School, Exeter, United Kingdom; 32 South Australian Health and Medical Research Institute, Adelaide, Australia

## Abstract

Coffee’s long-term effect on cognitive function remains unclear with studies suggesting both benefits and adverse effects. We used Mendelian randomization to investigate the causal relationship between habitual coffee consumption and cognitive function in mid- to later life. This included up to 415,530 participants and 300,760 coffee drinkers from 10 meta-analysed European ancestry cohorts. In each cohort, composite cognitive scores that capture global cognition and memory were computed using available tests. A genetic score derived using *CYP1A1/2* (rs2472297) and *AHR* (rs6968865) was chosen as a proxy for habitual coffee consumption. Null associations were observed when examining the associations of the genetic score with global and memory cognition (β = −0.0007, 95% C.I. −0.009 to 0.008, P = 0.87; β = −0.001, 95% C.I. −0.005 to 0.002, P = 0.51, respectively), with high consistency between studies (P_heterogeneity_ > 0.4 for both). Domain specific analyses using available cognitive measures in the UK Biobank also did not support effects by habitual coffee intake for reaction time, pairs matching, reasoning or prospective memory (P ≥ 0.05 for all). Despite the power to detect very small effects, our meta-analysis provided no evidence for causal long-term effects of habitual coffee consumption on global cognition or memory.

## Introduction

Coffee is one of the most widely consumed beverages worldwide^1^. Its health benefits and risks have long been debated, with some studies suggesting reductions in the risk of various diseases^2–5^ and mortality^6–8^, while other studies have suggested a potential increase in cardiovascular risk^9^.

Comprised of over 1,000 chemical compounds, coffee gains its popularity through its acute stimulatory effects, which are attributed to the pharmacological activity of caffeine^1^. Caffeine can readily cross the blood-brain barrier, and is believed to exert its neurocognitive effects by antagonizing adenosine receptors in the central nervous system^1^. Large-scale meta-analyses of prospective studies suggested that higher coffee consumption is associated with a lower risk for Alzheimer’s disease^10,11^, while a recent genetic study using summary data from large consortia did not support a causal association^12^. Studies on the effects of coffee consumption on specific cognitive domains have yielded mixed results for executive function and memory, with some suggesting benefits, and others showing null or adverse effects^13–15^. Inconsistent findings may reflect methodological problems commonly seen with dietary exposures including confounding and reverse causation, the latter being particularly important in this case given that caffeine intake is typically one of the first behaviors to be altered when the individual’s health status declines^16^.

Randomised clinical trials are the gold standard for testing causal effects, however, these are expensive to undertake and it is difficult to examine influences of prolonged exposures. An alternative is Mendelian randomization, also called “nature’s randomised trial”, which is a non-invasive and cost-effective observational approach to identify possible causal associations^17^. In Mendelian randomization, causality is inferred from the association between exposure-related genetic variants and the outcome of interest^18,19^. As genotypes are assigned (at random) at conception they will not be generally affected by disease or social and lifestyle factors, allowing us to overcome some of the common problems with observational epidemiology. For a commonly used addictive stimulant such as caffeine, which has been proposed to have both adverse and beneficial effects on cognitive outcomes^14,20^, Mendelian randomization is therefore an attractive approach to use as it allows us to address issues both in relation to safety and preventative potential.

Genome-wide association studies to date have identified 8 loci influencing habitual coffee intakes^21,22^. Two common genetic variants have the strongest effects and known biological mechanisms, one located between the cytochrome P450 1A1 (*CYP1A1*) and *CYP1A2* gene regions (rs2472297) and the other 51 kb upstream the aryl-hydrocarbon receptor (*AHR*) gene (rs6968865)^23^. CYP1A2 accounts for ~95% of hepatic caffeine clearance, and AHR plays a key role in metabolizing xenobiotics and inducing the transcription of *CYP1A2*^24^. In this study, including individual level data for up to 415,530 European ancestry participants, we examined the causal association between genetically instrumented habitual coffee intake, and cognitive function in mid- to later-life, with the aim of establishing causal evidence for potential long-term cognitive effects of coffee consumption. Given the variants influencing patterns of coffee consumption also affect caffeine intake and clearance^23–25^, secondary analyses were conducted with respect to habitual tea and caffeine intakes.

## Methods

### Study population

Data came from 10 European cohorts including the 1958 British birth cohort (1958BC), UK Biobank, Mothers of Avon Longitudinal Study of Parents and Children (ALSPAC-M), Northern Finland Birth Cohorts 1966 (NFBC1966), Cardiovascular Risk in Young Finns Study (YFS), Helsinki Birth Cohort Study (HBCS), Prospective Investigation of the Vasculature in Uppsala Seniors (PIVUS), Uppsala Longitudinal Study of Adult Men (ULSAM), Swedish twin registry (STR) and TwinGene studies. Participants in each study have provided an informed consent, and each study is covered by ethical approvals from the relevant ethics committees. A more detailed description of each cohort is provided in the supplementary text. Data analysis in each cohort was restricted to participants with complete information on *AHR* and *CYP1A1/2* genetic variants, who also had information on habitual coffee intake and cognitive measures.

### Genetic variants

Genome-wide association studies to date have identified eight loci influencing habitual coffee intake patterns^21,22^. For our primary analyses we chose the two strongest loci that have well-characterized biological functions affecting caffeine metabolism, *CYP1A1/2* and *AHR*^24^. The remaining six loci (*POR, EFCAB5, GCKR*, *ABCG2*, *MLXIPL*, and *BDNF*) were used in secondary analyses. Our strategy for the selection of instrumental variables is expanded upon in Supplementary Fig. S1.

The primary variant for *CYP1A1/2* was rs2472297, and for *AHR* rs6968865. In cohorts where rs6968865 was unavailable, its proxy, rs6968554 (r^2^ = 1.0) or rs4410790 (r^2^ = 0.97) was used (Supplementary Table S1). As the two variants had very similar effects on habitual coffee intake in the original GWAS^21^ we used an unweighted genetic score constructed by summing the number of intake-increase alleles in *CYP1A1/2* (T in rs2472297) and *AHR* (T in rs6968865; G in rs6968554; C in rs4410790)^23^. Information on genotyping and related quality control is provided in the supplementary text (Supplementary Table S1). In secondary analyses we used rs17685, rs9902453, rs1260326, rs1481012, rs7800944, and rs6265 for *POR, EFCAB5, GCKR*, *ABCG2*, *MLXIPL*, and *BDNF*, respectively.

### Habitual coffee, tea and caffeine consumption

Coffee intake was the primary exposure of interest, with tea and caffeine consumption only used in the secondary analyses. Habitual coffee consumption (cups/day) in each cohort was computed using the available self-reported intake information, and in cohorts where the intake was indicated in an interval (e.g. 2 to 4 cups a day), the median was used. A categorical indicator for the intake level (<1, 1–4 and ≥4 cups/day) was also created for the purpose of stratification in subgroup analyses. Habitual tea consumption (cups/day) was computed the same way as the habitual coffee consumption, and habitual caffeine intake (mg/day) was approximated by combining information on coffee and tea consumption and assuming that one cup of coffee and tea contained approximately 75 mg and 40 mg of caffeine, respectively^23^. Information on other caffeinated drinks (e.g. energy drinks, hot chocolate) was not available for most of the studies, and hence was not included in the computation for habitual caffeine intake. Detailed descriptions for coffee and tea intake information in each cohort can be found in the supplementary text (Supplementary Table S2).

### Global cognition and memory scores

Cognitive measures included in the cohorts ranged from tests of global cognition such as the Mini-Mental State Examination (MMSE) to domain specific tests such as the Trail Making Tests, Digit Symbol Coding and the Paired Associates Learning test (Supplementary Table S3). Given the diversity of cognitive measures across cohorts, we computed composite global cognition and memory scores and used them as our primary outcomes. Those composite scores were constructed within each cohort by summing the standardized scores of the relevant cognitive tests. Such composite scores are regularly used as a way of producing a more stable representation of cognitive function, harmonizing outcomes between cohorts, and maximizing interpretability^26^. Individual cognitive tests used in the global cognitive and memory scores were all coded to have higher scores as indicative of better cognitive function. Aside from the composite cognitive scores in the primary analyses, domain-specific cognitive measures in the UK Biobank, including reaction time (N = 288,905), pairs matching (N = 290,574), reasoning (N = 93,512) and prospective memory (N = 95,340) were used in the secondary analyses for domain-specific effects.

### Statistical analyses

Three primary association analyses were performed, including (1) the phenotypic associations between the habitual coffee intake patterns and global cognition and memory function, (2) genetic associations between the genetic score indexing higher coffee intakes and global and memory cognition, and (3) instrument validation assessing the association between the genetic instruments and coffee intake patterns. For the genetic associations, since the coffee-cognition relationship may vary by intake level, we performed the association analyses under different levels of habitual coffee intake (1–4 and ≥4 cups/day). As a negative control, we also examined the genetic associations among non-coffee drinkers (<1 cup/day). For all three association analyses we used the linear regression and fitted two models, with model 1 including sex and age as covariates, and model 2 adjusted for sex, age, smoking (non-current and current smokers), education attainment and depression. In the genetic analyses we assumed an additive inheritance model, and additionally adjusted for principal components (PCs) to account for population stratification.

We performed secondary subgroup analyses to test if the genetic and phenotypic associations varied by age (<65 or ≥65 years) or sex. Since the genetic instruments indexing habitual coffee intake likely influence the intake patterns through affecting caffeine clearance^23–25^, we conducted secondary analyses for habitual tea and caffeine intakes (similar to the analyses performed for habitual coffee intake), with analyses on tea consumption further restricting the data to participants who did not drink coffee. Further, in the UK Biobank with a sample size up to 290,574, we performed domain-specific analyses to complement our primary findings using composite cognitive scores. Additional sensitivity analyses were conducted using alternative genetic instruments (*POR* and *EFCAB5*) to index habitual coffee intakes. Furthermore, we conducted analyses using an alternative MR approach, MR-Egger regression, which is suggested to be robust to pleiotropy^27^ and which therefore allowed us to use all eight genome-wide significant variants associated with habitual coffee intake patterns (*CYP1A1/2, AHR, POR, EFCAB5, GCKR*, *ABCG2*, *MLXIPL*, and *BDNF*)^21^.

Data analyses were conducted in each participating cohort, and the results were sent to the University of South Australia for quality control and meta-analyses. To ensure the uniformity in data analysis across cohorts, a detailed analysis plan and related statistical script was supplied to all cohorts. Random-effects models were used for the meta-analyses, with sensitivity analyses using fixed effects in the absence of heterogeneity. Meta-regression was used to assess variation by cohort characteristics, including age, sex or country. Further, power analyses^28^ were performed to determine the minimal effect size we were powered to detect in the primary and domain specific genetic association analyses. All data analyses were performed using Stata (StataCorp LP, College Station, Texas, USA).

## Results

Information for coffee intake was available for 415,530 participants from 10 European cohorts, including 300,760 coffee consumers. Basic characteristics for participants in different studies are shown in Table 1. Median daily coffee consumption ranged from 2 to 4 cups/day (Table 1), with more coffee being consumed daily in Finland and Sweden than in the UK. Allele frequencies for *AHR* and *CYP1A1/2* were consistent with those reported for the respective populations in the 1000 Genomes project. The genotype frequencies of both variants were in Hardy-Weinberg equilibrium in all cohorts (P > 0.07).

**Table 1:** Characteristics of the participating cohorts.

	Country	N^*^	Males (%)	Age, yrs		Coffee intake, cups/day			Global scores Median (IQR)	Memory scores Median (IQR)	*AHR* MAF (%)	*CYP1A1/2* MAF (%)
				≥65 yrs (%)	Median (IQR)	1–4 cups/day (%)	≥4 cups/day (%)	Median (IQR)				
1958BC	UK	4,416	50.7	0.0	50 (0)	48.1	18.6	3 (1)	0 (1.3)	0.04 (1.3)	38.7	26.4
ALSPAC-M	UK	4,734	0.0	0.0	51 (6)	53.1	21.3	2 (3)	0.03 (1.4)	0.13 (1.3)	36.2	26.5
UK Biobank	UK	404,620	46.0	19.6	58 (12)	51.4	20.4	2 (3)	0.2 (1.1)	0.34 (1.2)	36.4	26.7
HBCS	Finland	1,653	41.5	90.0	68 (4)	73.2	17.0	2.5 (0.4)	0.08 (1.3)	0.12 (1.3)	35.6	19.6
NFBC1966	Finland	3,488	43.5	0.0	47 (1)	32.2	63.4	4 (3)	−0.06 (1.2)	−0.06 (1.2)	31.6	24.6
YFS	Finland	2,323	45.7	0.0	43 (9)	38.2	44.1	4 (3)	0.14 (1.3)	0.22 (0.9)	31.4	25.0
PIVUS	Sweden	882	49.9	100.0	70 (0)	62.4	30.1	3.1 (1.9)	0.18 (1)	0.17 (1.2)	35.9	28.7
ULSAM	Sweden	1,081	100.0	100.0	71 (1)	63.2	32.4	3.4 (1.9)	−0.16 (1.2)	—	34.7	26.8
STR	Sweden	1,073	45.0	76.1	72 (10)	45.3	51.1	4 (2)	0.05 (1.4)	−0.09 (1.5)	37.7	28.7
TwinGene	Sweden	2,370	51.3	100.0	69 (6)	53.9	40.1	3 (2)	0.09 (1.2)	0.16 (1.2)	34.8	27.7

IQR: interquartile range; MAF: minor allele frequency. ^*^Numbers reflect the maximal sample of individuals from each cohort who had genetic data.

### Phenotypic associations

There was no evidence for an overall phenotypic association between habitual coffee intake and global cognition or memory function (Table 2). Although higher coffee intake appeared to be associated with lower global cognitive scores in the sex and age adjusted analyses (Table 2, P = 0.04), this tentative association was no longer present after further adjustment for smoking, education level and depression (Table 2, P = 0.26).

**Table 2:** Phenotypic and genetic associations of habitual coffee intake with cognition.

	Global cognition					Memory cognition				
	N	β* (95% C.I.)	P	I^2^	P_heterogeneity_	N	β* (95% C.I.)	P	I^2^	P_heterogeneity_
Phenotypic effect (cups/day)	300,806	−0.02 (−0.039, −0.001)	0.04	85	<0.001	301,850	−0.012 (−0.029, 0.005)	0.16	80	<0.001
Adjusted phenotypic effect (cups/day)	295,823	−0.008 (−0.023, 0.006)	0.26	74	<0.001	296,777	−0.004 (−0.017, 0.01)	0.58	66	0.003
Genetic effect (intake-increase-allele)^#^	300,760	−0.0007 (−0.009, 0.008)	0.87	4	0.40	301,804	−0.001 (−0.005, 0.002)	0.51	0	0.64

^*^All models adjusted for sex and age, with the adjusted phenotypic model controlling further on smoking, education and depression and the genetic models additionally adjusting for principal components. ^#^Among coffee drinkers.

There was considerable variability in the phenotypic associations across studies, with I^2^ ranging from 66% to 85% (P_heterogeneity_ ≤ 0.003 for all comparisons, Table 2). However, there were no systematic study level differences in the phenotypic associations by age, sex or country (P_meta-regression_ > 0.05 for all).

### Genetic associations and instrument validation

Genetic associations were not affected by adjustment for smoking, education level and depression. Results are presented for the simpler model with adjustment for sex, age, and PCs to minimize the risk of collider bias potentially introduced by the statistical adjustment^29^.

**Figure 1:**
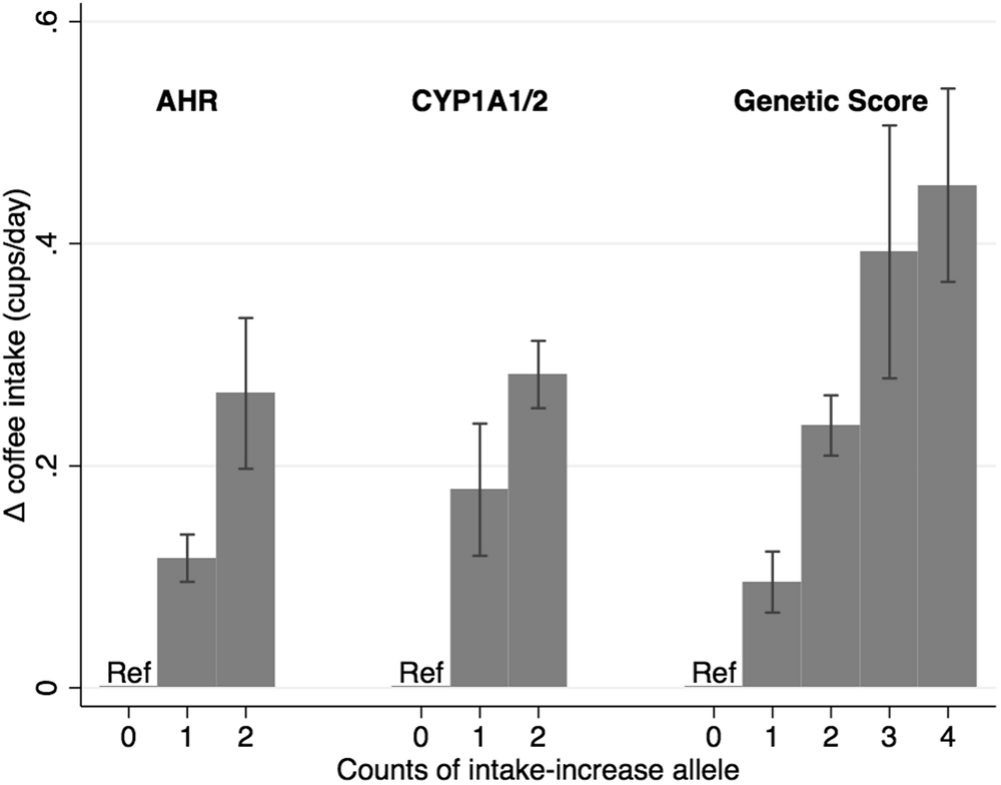
Association of *AHR*, *CYP1A1/2* and genetic score with habitual coffee intake (cups/day) among coffee drinkers. Error bars are the 95% confidence intervals.

*CYP1A1/2*, *AHR* and the genetic score showed the expected patterns with habitual coffee consumption (Fig. 1), with each allele on the genetic score associated with an average of 0.16 additional cups of coffee/day (95% C.I. 0.12–0.20, P = 3 × 10^−15^).

**Figure 2:**
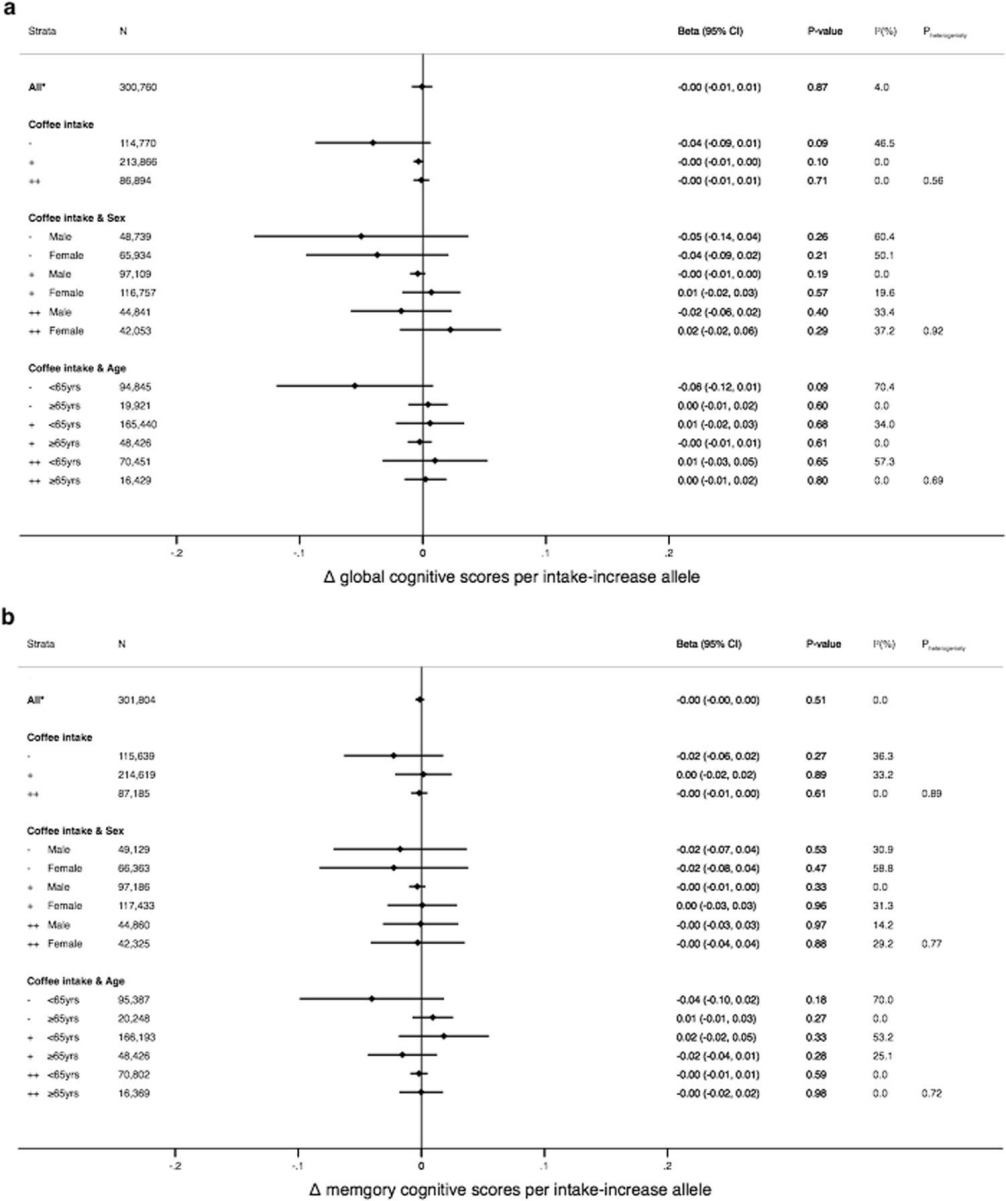
Association of genetic score with global (**a**) and memory (**b**) cognition among coffee drinkers and in subgroups stratified by coffee intake (<1, 1–4 or ≥4 cups/day), sex and age (<65 yrs or ≥65 yrs). *Among coffee consumers; −, + and ++ denote <1, 1–4 and ≥4 cups/day, respectively. Error bars are the 95% confidence intervals.

There was no evidence for an association between the genetic score with global cognition or memory among habitual coffee drinkers (P > 0.5 for both, Fig. 2), with high consistency between the cohorts (I^2^ ≤ 4% for both, Table 2 and Supplementary Fig. S6). Further, there was also no evidence for variation in the genetic effect by the degree of habitual coffee intake (Fig. 2, P_heterogeneity_ ≥ 0.56 for both global cognition and memory). As expected, in analyses restricted to participants who did not drink coffee as the negative control, the genetic score was not associated with global cognition or memory (P > 0.09 for both, Fig. 2).

### Secondary analyses

**Figure 3:**
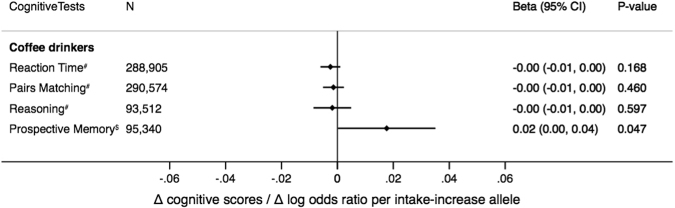
Association of the genetic score with different cognitive tests among coffee consumers in the UK Biobank. For all cognitive tests, positive effect sizes indicate improvements in cognitive function. For the prospective memory (i.e. a binary outcome), odds = probability of being correct/probability of being incorrect at the first attempt. Error bars are the 95% confidence intervals. ^#^Effect size is change in cognitive scores per intake-increase allele; ^$^Effect size is change in log odds ratio per intake-increase allele.

Phenotypic and genetic effects did not vary by age (<65 yrs or ≥65 yrs) or sex (P_heterogeneity_ > 0.19 for all comparisons). Sensitivity analyses using two additional variants associated with coffee consumption, *POR* and *EFCAB5*^21^, provided no consistent evidence for association with global or memory cognition (Supplementary Fig. S2). An isolated association with memory function was seen for *EFCAB5* (P = 0.004), a variant which had previously been suggested to be “non-pleiotropic” indicator for coffee consumption^12^, but which is located near the serotonin transporter^21^. MR-Egger regression, using all eight genome-wide significant variants associated with habitual coffee intake^21^, also provided no evidence for association with global or memory cognition (−0.0096 [95% C.I. −0.056 to 0.036], and 0.0054 [95% C.I. −0.048 to 0.058], respectively). Domain specific information was available in the UK Biobank for reaction time (N = 288,905), pairs matching (N = 290,574), reasoning (N = 93,512) and prospective memory (N = 95,340). The genetic score was not associated with reaction time, pairs matching, or reasoning among coffee drinkers (P ≥ 0.05 for all, Fig. 3). For prospective memory the results were inconclusive (P = 0.047), however, this nominal benefit was not consistently seen in across caffeine intake variants (Supplementary Fig. S3).

Intake-increase alleles of *CYP1A1/2*, *AHR* and the genetic scores associated with increased habitual tea and caffeine intake in a dose-dependent matter (Supplementary Fig. S4). We examined the association of the genetic score with cognition among tea/caffeine consumers, including corresponding subgroup analyses, with null associations found in all these analyses (Supplementary Fig. S5). Sensitivity analyses on tea consumption restricting data to participants who did not drink coffee (N = 103,064) also obtained null associations (P = 0.35 and 0.57 for global cognition and memory function, respectively). Detailed results can be found in the supplementary text.

### Instrument validation in the UK Biobank

In the UK Biobank, the genetic score explained 0.33% of variation in coffee consumption patterns (F-statistic = 952.6). There were no significant imbalances in allele distribution of *CYP1A1/2*, *AHR* or genetic score by background covariates (P ≥ 0.05 for all, Supplementary Table S5).

### Power calculations

For the genetic association, with a sample size of 300,760 (the sample size for global cognitive function) and a two-sided α level of 0.05, at 80% of power we would sufficiently detect a 0.0055 standard deviation (SD) change in the cognitive score for every coffee-intake-increase allele. Approximating from the distribution of coffee intake in the UK Biobank (SD = 2 cups/day), this translates into a causal effect of 0.045 SD by each cup of coffee. For the domain specific analyses in the UK Biobank, with the lowest sample size of 93,512 (the sample size for the reasoning test), the effect size that we would be sufficiently powered to detect is 0.0099 SD for a continuous outcome and 0.024 log odds ratio (log OR, OR 1.02) for a binary outcome. This translates into a 0.081 SD and 0.20 log OR (OR 1.22) by each cup of coffee, respectively.

## Discussion

Our large-scale genetic analyses including over 300,000 coffee drinkers did not find any evidence to support beneficial or adverse long-term effects of coffee intake on global cognition or memory. Our findings are in line with a recent Mendelian randomization study on Alzheimer’s disease^12^, but contrasts with earlier observational studies^14^, alleviating concerns about potentially adverse effects on memory function. Our null finding is convincing given the power to detect even very small effects, and as the results were remarkably consistent between cohorts, and also across different genetic variants used to index habitual coffee intake. While our analyses did not provide evidence for long-term benefits, these data suggest that there are no adverse effects on memory or cognitive function.

Mendelian randomization has become an increasingly popular approach for examining causal relationships, and it has begun to show its potential by clarifying a number of previously misconceived associations^30,31^. For studies to investigate the effects of coffee intake, it has the obvious attraction in the ability to test the preventative potential before advancing to more costly intervention studies. However, as used in the present study, Mendelian randomization has great prospects in addressing safety concerns with exposures considered to be potentially harmful.

Global cognitive function encompasses several domains with each domain itself being a complex entity^32^. It is possible that the effect of habitual coffee consumption may vary from one domain to another. If this is true, then composite cognitive scores capturing different cognitive domains may potentially dilute any domain-specific effects. Domain-specific analyses were conducted using smaller sub-samples of the UK Biobank with available information, with no evidence for either benefit or harm by coffee intake. Some uncertainty remains with prospective memory, where results were borderline, retaining the possibility of some nominal benefit. Given that this effect was not consistently observed across all instruments for habitual coffee intake it is most likely to be a chance finding.

In Mendelian randomization the ability to make causal inferences directly depends on the availability and quality of genetic instruments. Genome-wide association studies to date have identified eight loci influencing habitual coffee intakes^21,22^, including *CYP1A1/2*, *AHR*, *POR*, *EFCAB5*, *GCKR*, *ABCG2*, *MLXIPL*, and *BDNF*. We chose to use the two strongest variants to construct our primary instrument, as both consistently associate with habitual coffee intake^21–23^ and have well-characterized biological mechanisms influencing caffeine metabolism which plausibly explain their association with coffee intake^24^. However, pleiotropic effects cannot be fully excluded given genome-wide association analyses on Parkinson’s disease, blood pressure and bladder cancer have shown signals both for *AHR* and *CYP1A1/2*^33–35^, although these weak associations could reflect downstream causal effects of caffeine or coffee consumption^36^. To exclude the possibility that the null association with cognitive function which we observed in this study is driven by opposing patterns induced by pleiotropy in *AHR* and/or *CYP1A1/2*, we conducted sensitivity analyses in the UK Biobank using other variants to index habitual coffee consumption^21^ and replicated our main analyses including *POR* and *EFCAB5*, which have unknown effects on caffeine metabolism but which did not have previously reported pleiotropic effects. Also these analyses provided no support for causal effects of habitual coffee intake on cognition, although there appeared to be an association between memory function and rs9902453, located in the intron of the *EFCAB5*. There is very limited information on *EFCAB5* in the literature, however, the variant is in close proximity to *SLC6A4*, the gene encoding serotonin transporter^21^. Serotonin affects cognitive abilities, in particular memory consolidation^37^, hence, the observed rs9902453–memory function association is likely a reflection of a pleiotropic effect than a true causal effect of coffee intake. Pleiotropy was also considered to be a problem with four other variants identified in the earlier GWAS on coffee consumption (notably *GCKR*, *ABCG2*, *MLXIPL*, and *BDNF)*^21^, which according to the GWAS catalogue, had shown primary associations with traits including BMI, smoking initiation, and gout^12^. In further sensitivity analyses we included all eight coffee intake associated variants^21^ in the MR-Egger regression which is robust to pleiotropy^27^. However, any change in our instrument selection strategy did not affect our conclusions, and also these analyses provided no support for any causal association between habitual coffee intake and cognition.

As secondary analyses, we examined the association of the genetic instruments with habitual tea and caffeine intakes. While people who consume more coffee tend to drink less tea, our analyses suggest that the genetic drivers for coffee and tea consumption are similar. This, together with the known functions of *CYP1A1/2* and *AHR*, confirm that influences on caffeine metabolism likely explains the association between the genetic instruments and habitual coffee intake. It can be argued as a limitation with our approach that the genetic instruments, which were used to index habitual coffee intake, can also index habitual tea intake. However, it is unlikely that a causal coffee-cognition relationship would have been confounded by habitual tea intakes in our study, given the notably weaker genetic influences on tea than on coffee consumption. Further, it needs to be noted that, any inferences relating to caffeine will need to be drawn with caution as the instruments indexing greater caffeine consumption may reflect a faster rate of caffeine clearance, and hence a lower (rather than higher) circulating levels of bioactive caffeine^38^.

As a further limitation, it can be argued that our genetic association results among coffee drinkers could have been affected by the collider bias as the genetic score indexing the extent of habitual coffee intake is also associated with the probability of being a coffee drinker^39^. However, sensitivity analyses in the UK Biobank study conducted in the coffee and non-coffee drinkers combined, also showed a null association (results not shown). Also, while the test for the association between the genetic score and the outcome is a valid test for a causal relationship^40^, it cannot estimate the magnitude of the causal effect if it exists. However, given that null associations were observed in our study, there would have been little gain in applying additional methods (such as instrumental variable analyses)^41^ to quantify these ‘null’ effects.

In conclusion, we found no evidence that habitual coffee consumption is causally associated with global and memory cognition in mid- to later-life, despite the power to detect very small effects. This suggests that interventions to protect cognition or slow cognitive decline using coffee are unlikely to be successful and should not be prioritized in future trials. That said, there was no evidence for any adverse effect, contrary to some previous observational studies, and hence it appears safe to consume coffee at least with respect to preserving memory function.

## Electronic supplementary material

Supplementary Material
