# Supplementary material for: Habitual coffee consumption and cognitive function: a Mendelian randomization meta-analysis in up to 415,530 participants
Source: Sci Rep. 2018 May 14;8:7526. doi: 10.1038/s41598-018-25919-2 (PMC5951917; doi:10.1038/s41598-018-25919-2)
Supplement: Supplementary file 1 — Supplementary Material [file 41598_2018_25919_MOESM1_ESM.docx]

**Habitual coffee consumption and cognitive function: a Mendelian randomization meta-analysis in up to 415,530** **participants**

Ang Zhou, Amy E Taylor, Ville Karhunen, Yiqiang Zhan, Suvi P. Rovio, Jari Lahti, Per Sjögren, Liisa Byberg, Donald M. Lyall, Juha Auvinen, Terho Lehtimäki, Mika Kähönen, Nina Hutri-Kähönen, Mia Maria Perälä, Karl Michaëlsson, Anubha Mahajan, Lars Lind, Chris Power, Johan G Eriksson, Olli T. Raitakari, Sara Hägg, Nancy L. Pedersen, Juha Veijola, Marjo-Riitta Järvelin, Marcus R Munafò, Erik Ingelsson, David J. Llewellyn & Elina Hyppönen

Supplementary Materials

1. Supplementary Methods 3

Study Description and Cognitive Measures 3

1958 British Birth Cohort (1958BC) 3

Avon Longitudinal Study of Parents and Children-Mothers (ALSPAC-M) 4

Helsinki Birth Cohort Study (HBCS) 5

Northern Finland Birth Cohort 1966 (NFBC1966) 6

The Prospective Investigation of the Vasculature in Uppsala Seniors (PIVUS) 7

Swedish Twin Registry (STR): GENDER, SATSA 8

TwinGene 9

UK Biobank 9

Uppsala Longitudinal Study of Adult Men (ULSAM) 10

Young Finns Study (YFS) 11

Table S1. Coffee-intake SNPs in each cohort 13

Table S2. Coffee intake information in each cohort 14

Table S3. Cognitive measures for global and memory cognition 15

Table S4. Depression, education and study-specific covariates in each cohort 16

2. Supplementary Results 18

Figure S1. Usage of genome-wide significant coffee variants as genetic instruments for habitual coffee intake. 18

Figure S2. Association of different genetic instruments with global (a) and memory (b) cognitive scores, among coffee drinkers in the UK Biobank. 19

Figure S3. Association of different genetic instruments with prospective memory, among coffee drinkers in the UK Biobank. 20

Figure S4. Association of *AHR*, *CYP1A1/2* and genetic score with habitual tea (a) and caffeine (b) intake. 21

Figure S5. Association of *AHR*, *CYP1A1/2* and genetic score with global and memory cognitive scores, among coffee, tea and caffeine consumers. 22

Figure S6. Association of genetic score with global (a) and memory (b) cognitive scores from the crude model, among coffee drinkers in individual cohort. 23

Figure S7. Association of genetic score with global (a) and memory (b) cognitive scores from the adjusted model, among coffee drinkers in individual cohort. 24

Table S5. Association of *AHR*, *CYP1A1/2*, and genetic score with habitual coffee intake in individual cohort 25

Table S6. Association of *AHR*, *CYP1A1/2* and genetic score with confounders in the UK Biobank study 26

3. ACKNOWLEDGEMENT 27

4. REFERENCES 29

# Supplementary Methods

## Study Description and Cognitive Measures

### 1958 British Birth Cohort (1958BC)

*Participants*

The 1958BC includes all births during one week in March in England, Scotland and Wales (~ 17,000) ^1^. Participants in the study have been followed regularly with information collected on a wide-range of factors related to health, lifestyle, growth and development. At age 45 years (August 2002 to March 2004) cohort members were invited to a biomedical survey, in which blood samples were taken and DNA were extracted for genotyping. Caffeine SNP genotypes were extracted from the genome-wide information, which were obtained through two sub-studies, WTCCC2 (n=3,000) ^2^ and T1DGC (n=2,592) ^3^, both using the 1958BC participants as controls. Self-reported habitual coffee and tea intake information were collected at the age of 45. Cognitive tests took place at 50 years survey.

*Cognitive tests*

Four cognitive tests were performed, including Immediate Word Recall ^4^, Delayed Word Recall ^4^, Verbal Fluency (Animal Naming) ^5^ and Letter Cancellation ^6^.

- Immediate Word Recall: In the test participants were asked to recall as many words as possible from a list of 10 common words read out by interviewer.
- Delayed Word Recall: In the test participants were asked to recall as many words as possible from a list of 10 common words read out by interviewer, following a short delay of approximately 5 minutes.
- Verbal Fluency (Animal Naming): This test measures how many words related to a category (in this case, types of animals) a participant can produce.
- Letter Cancellation: This test measures speed of processing. Participants were instructed to cross out as many target letters (P and W, 65 in total) as possible in one minute.

### Avon Longitudinal Study of Parents and Children-Mothers (ALSPAC-M)

*Participants*

ALSPAC recruited 14,541 pregnant women resident in Avon, UK with expected dates of delivery 1st April 1991 to 31st December 1992 ^7,8^. When the oldest children were approximately 7 years of age, an attempt was made to bolster the initial sample with eligible cases, who had failed to join the study originally. The children, their mothers and their partners have been followed up ever since through questionnaires and clinic invitations. In the current study, only mother’s data were used in the data analysis. Only unrelated individuals of European descent were included in the analysis. Coffee and tea intake information were taken from a questionnaire administered to mothers when the study children were aged 12 years. Please note that the study website contains details of all the data that is available through a fully searchable data dictionary" and reference the following webpage: http://www.bris.ac.uk/alspac/researchers/data-access/data-dictionary. Ethical approval for the study was obtained from the ALSPAC Ethics and Law Committee and the Local Research Ethics Committees.

*Genetic data*

Genotyping of 10,015 of the ALSPAC mothers was carried out on the Illumina human660W-quad array by the Centre National de Genotypage. In total 557,124 SNPs were directly genotyped. Quality control measures were carried out using PLINK (v1.07) ^9^. SNPs were removed based on missingness (>5%), an HWE p-value of <1.0e-06 or a minor allele frequency of <1%. Samples were removed on the basis of missingness (>5%), indeterminate X chromosome heterozygosity or high autosomal heterozygosity. Samples were restricted to Europeans and unrelated individuals (cryptic relatedness >0.125). Imputation was performed using Impute V2.2.2 against the 1000 genomes reference panel (Phase 1, Version 3). After quality control, 8196 mothers with genetic data were available for analysis.

*Cognitive test*

Cognitive measures were taken from “Focus on Mothers 2” clinic, which took place between 2011 and 2013. The participants underwent a series of six cognitive tests, including Logical Memory 1 and 2 ^10^, Backwards Digit Span ^11^, Digit Symbol Coding ^11^, Spot-the-Word Test ^12^ and Verbal Fluency Test ^13^.

- Logical Memory 1: A standardized recording of a short story was played to the participants, who were asked to listen carefully and try to remember it the way it was said. After playing the story, participants were asked to tell the fieldworker everything they could remember about the story.
- Logic Memory 2 – delayed recall: After completing all other cognitive tests participants were asked to recall the story, which was told in the logic memory 1 test.
- Backwards Digit Span: Fieldworkers gave a series of numbers out loud and asked the participants to recall them backwards with no time for pause.
- Spot-the-Word Test: Participants were given a series of sixty pairs of words. Each pair contained one real and one nonsense word. Participants were asked to place a tick next to the word in each pair that they thought was the real word.
- Digit Symbol Coding: Participants were shown a series of symbols, each of which was associated with a number from 1 to 9. They were then asked to fill in a grid drawing the correct symbol next to each number one after the other without skipping any.
- Verbal Fluency Test: Participants were given a letter of the alphabet and asked to say as many words as they can think of that beginning with that letter. They were not allowed to include proper nouns, (people’s names or towns or numbers, or any word which would have a capital letter). They were also not allowed to include the same word with a different ending. A practice was given with the letter ‘S’. After that 1-minute periods were timed for the letters ‘C’, ‘F’ and ‘L’. Participants were scored one point for each correct entry done in the allocated time. A total score was obtained by summing all three scores.

### Helsinki Birth Cohort Study (HBCS)

*Participants*

The HBCS is a longitudinal study focused on the early origins of health and disease ^14^. Information for this study was collected using data from the maternity hospital, clinical, school databases and other registries on people born in 1934-1944 in two hospitals in Helsinki Finland. During 2001-2003, 2,003 men and women participated in the clinical follow-up study, in which blood samples were collected and cognitive testing was administered.

*Cognitive tests*

HBCS uses CERAD (The Consortium to Establish a Registry for Alzheimer's disease) to assess participants’ cognitive functions. CERAD consists of the following tests:

- Verbal Fluency, Animal Naming: It measures how many words related to a category (in this case, types of animals) a participant can produce.
- Immediate Verbal Recall: Participants were asked to recall as many words as possible from list of 10 words in 90 seconds.
- Delayed Verbal Recall: Participants were asked to recall as many words as possible from list of 10 words after a delay.
- Verbal Learning: Participants were shown a list of 10 words 3 times then asked to recall as many words as possible. Total score is the number of correct words recalled.

### Northern Finland Birth Cohort 1966 (NFBC1966)

*Participants*

NFBC 1966 was initiated in 1965 by enrolling mothers living in the two Northernmost provinces of Finland (Oulu and Lapland) and with expected dates of delivery in 1966 ^15^. A total of 12231 children were born into the cohort, 12058 of them live-born. Baseline data have been supplemented by data collected with postal questionnaires at the ages of 1, 14, 31 and 46 years and various hospital records and national register data. In 1997, those still living in the original target area (Northern Finland) or in the capital (Helsinki) area were invited to a clinical examination, in which 71% (n=6033) participated. Blood samples were drawn and DNA was extracted successfully for 5753 of these subjects. In 2012, all cohort members alive and with known address in Finland (n=10321) were invited for a 46-year follow-up, including postal questionnaire and clinical examination. Questionnaire data was obtained for 6868 subjects (66.5 %) and clinical examination data for 5861 subjects (56.7 %).

*Cognitive tests*

- Paired Association Learning Test: This test assesses visual memory and new learning

### The Prospective Investigation of the Vasculature in Uppsala Seniors (PIVUS)

*Participants*

The PIVUS study started in 2001 with the primary aim to investigate the predictive power of different measurements of endothelial function and arterial compliance in a random sample of 1,000 subjects aged 70 living in the community of Uppsala ^16^. In March 2006 a re-investigation of the cohort at the age of 75 was started. Cognitive tests were included in the re-investigation that was completed in September 2009. Of the initial 1016 subjects, 52 had died during the 5 years and 827 attended the re-examination at age 75.

*Cognitive tests*

Cognitive tests included the Mini–Mental State Examination (MMSE), Swedish translation of the Seven Minute Screening (7MS) Test, Trail Marking Test (TMT) A and TMT B.

- MMSE ^17^: 30-point questionnaire was used to screen for cognitive impairment
- 7MS test ^18^: The test consisted of (1) Benton temporal orientation i.e. measurement of orientation in time (2) Clock drawing i.e. subject draws the face of a clock and places the hands on a fixed time (3) Verbal fluency i.e. participant names as many different animals as possible in one minute (4) Enhanced cued recall i.e. identification and recall of 16 pictures immediately and after an interval with semantic cues if necessary.
- TMT: Participants were asked to draw lines with a pencil between the numbers in the right order as fast as possible. The score is equal to the time in seconds. TMT A consists of digits 1-25. TMT B consists of digits and letters 1-A-2-B etc.

### Swedish Twin Registry (STR): GENDER, SATSA

*Participants*

The participants were taken from longitudinal twin studies of aging. All these studies have previously been described in detail and are sub-samples of the population based Swedish Twin Registry ^19-21^. The Swedish Adoption/Twin Study of Aging (SATSA) ^21^ with up to six longitudinal occasions and the GENDER study ^20^ with two interview occasions. All participants are Caucasians and born in Sweden. For these sub-samples, individuals participated in at least one in-person session and in which blood samples were drawn.

*Cognitive tests*

Memory cognition for SATSA consisted of immediate word recall and delayed word recall, whereas in the GENDER study Thurstone's memory test was used as memory cognition. Global cognition in both studies came from the first component of cognitive measurement scale.

- Immediate Word Recall
- Delayed Word Recall
- Thurstone’s Memory Test: Subjects are shown 28 pictures and then asked for recognition of these among others. The pictures were enlarged from the original version to minimize any possible visual problems.
- General cognitive ability was calculated from principal component analysis of four tests (Synonyms, Block Design, Thurstone Picture Memory and Symbol Digit in GENDER, and from constructing the first principal component of nine cognitive tests in SATSA (Analogies, Synonyms, Information, Block Design, Card Rotations, Digits Span (Forward and Backward), Thurstone Picture Memory, Symbol Digit, and Figure Identification).

### TwinGene

*Participants*

The TwinGene project, conducted between 2004 and 2008, is a population-based Swedish study of twins born between 1911 and 1958 ^22^. The study participants have previously participated in a telephone interview called Screening Across the Lifespan Twin Study, conducted between 1998 and 2002. To be included in TwinGene, both twins within a pair had to be alive. The zygosity of the twins was based on self-reported childhood resemblance, or by using DNA markers (for 18% of the total sample). In total, 12591 individuals participated by donating blood to the study, and by answering questionnaires about life style and health. The study was approved by the local ethics committee at Karolinska Institute and all participants gave informed consent.

*Cognitive tests*

Memory cognition includes immediate word recall and delayed word recall. Global cognition was constructed using a cognitive screening score (<https://dornsife.usc.edu/assets/sites/342/docs/Tele.pdf>) constructed from Immediate Word Recall, Delayed Word Recall and Similarity Test.

### UK Biobank

*Participants*

The UK Biobank is a large prospective study with over 500,000 participants from across the United Kingdom and aged 40–69 years at recruitment in 2006–2010 ^23^. The study has both data from questionnaires, physical measures, sample assays, accelerometry, multimodal imaging, genome-wide genotyping and longitudinal follow-up for a large number of health-related outcomes. We have restricted the analyses to individuals, who are genetically defined as of European descent, and excluded those with mismatched information between self-reported and genetic sex. Relatedness for between participants (2^nd^ degree or closer) was accounted by using probability weights ^24^, with weights assigned as 1 – kinship coefficient ^25^. Two individuals were allowed from each family, with the pair having the lowest kinship coefficient retained in the analyses.

*Cognitive tests*

In the current study, pairs matching and reaction time tests were used in our primary analyses for the construction of global and/or memory cognitive scores, whereas reasoning and prospective memory tasks, which were available to a subsample of participants, were only used in the secondary analyses to test domain specific effects. Cognitive tests in the UK Biobank have been described previously ^26,27^.

- Pairs Matching Test: Participants were asked to memorize the position of as many matching pairs of cards as possible. The cards are then turned face down on the screen and the participant is asked to touch as many pairs as possible in the fewest tries.
- Reaction Time: It is a test, based on 12 rounds of the card-game 'Snap' to assess one’s reaction time. In the test, participants were shown two cards at a time. If both cards were the same, they were asked to press a button-box in front of them as quickly as possible.
- Reasoning: Participants were asked to complete as many questions related to 'fluid intelligence' (i.e. the capacity to solve problems that require logic and reasoning ability, independent of acquired knowledge) as possible within 2 minutes.
- Prospective Memory: In the beginning of the test the participants were shown the message "At the end of the games we will show you four coloured shapes and ask you to touch the Blue Square. However, to test your memory, we want you to actually touch the Orange Circle instead." We dichotomized the data to either ‘correct on first attempt’ or not.

### Uppsala Longitudinal Study of Adult Men (ULSAM)

*Participants*

Men born between 1920 and 1924 in Uppsala, Sweden were invited to participate at age 50 (N=2,841) in this longitudinal cohort study, which was started in 1970 ^28^; 81.7% (N=2,322) participated. Subjects were re-investigated at the ages of 60, 70, 77, 82 and 88 years.

*Cognitive tests*

Cognitive tests comprised of Mini–Mental State Examination (MMSE) and Trail Marking Test (TMT).

- MMSE ^17^: 30-point questionnaire used to screen for cognitive impairment
- TMT ^29^: Participant is asked to draw lines with a pencil between the numbers in the right order as fast as possible. The score is equal to the time in seconds. TMT A consists of digits 1-25. TMT B consists of digits and letters 1-A-2-B etc.

### Young Finns Study (YFS)

*Participants*

The Cardiovascular Risk in Young Finns Study (YFS) is an ongoing longitudinal population-based study focusing on cardiovascular risk factors from childhood to adulthood. The study was originally designed as a national collaborative effort between all university hospitals and several other institutions in Finland. The first cross-sectional study of the YFS was performed in 1980, and it included 3,596 randomly selected children and adolescents (both boys and girls) aged 3, 6, 9, 12, 15 and 18 years. Until the year 2011, the cohort has been regularly followed-up in 3-9 year intervals. More detailed information on the YFS study population and protocol is reported elsewhere ^30^. Blood samples were collected in the 2007 wave and cognitive tests were conducted in 2011.

*Cognitive tests*

- Paired Associates Learning Test: During this test either 1, 2, 3, 6, or 8 patterns were displayed sequentially in boxes placed on the screen. After that, the patterns were presented in the center of the screen, and the participants were supposed to point the box in which the particular pattern was previously seen. The test moves on to the next stage if all the patterns were placed to right boxes. In case of incorrect response, all the patterns were re-displayed in their original locations and another recall phase was followed. The test terminated if the patterns were still incorrectly placed after 10 presentation and recall phases.
- Spatial Working Memory Test: During this test the participants were presented with randomly distributed colored boxes ranging in number from 4 to 8. After that the participants were supposed to search for tokens hidden in the boxes. When a token was found it was supposed to be moved to fill an empty panel on the right-hand side of the screen. Once the token had been moved from the box, the participant had to recall that the computer would never hide a new token in a box that previously contained one; therefore the participants were not supposed to revisit the same boxes again.
- Reaction Time Test: In the first part of the test, a large circle was presented in the centre of the screen. The participant was supposed to press a button on a press pad until a small yellow spot appears in the large circle. When the yellow spot appeared the participant was supposed to touch the spot as soon as possible with the same hand that was pressing the button on the press pad. In the second part of the test, the same task was performed, except that in this part five large circles were presented on the screen, and the small yellow spot might appear in any of the five circles. Again the participant was supposed to touch, as soon as possible, the yellow spot with the hand pressing the button on the press pad.
- Rapid Visual Information Test: In the test the participant was presented with a number sequence (e.g. 3, 5, 7) next to a large box where numbers appeared in a random order. Whenever the particular sequence was presented, the participant was supposed to press a button on a press pad. At the beginning, the participant was given visual cues (i.e. colored or underlined numbers) to help to recognize the particular sequence. When the test proceeded, the cues were removed.

## Table S1. Coffee-intake SNPs in each cohort

|  |  | ***CYP1A1/2*** | | | ***AHR*** | | |
| --- | --- | --- | --- | --- | --- | --- | --- |
| **Study** | **Platform** | **SNP ID** | **Directly genotyped / imputed** | **HWE, P** | **SNP ID** | **Directly genotyped / imputed** | **HWE, P** |
| **1958BC** | Illumina 550k for T1DGC Affymetrix 6.0 for WTCCC2 | rs2472297 | Directly genotype  Call rate: > 95% | 0.70 | rs6968865 | Imputed  Software: IMPUTE  Reference panel: HapMap R22, CEU  Quality: proper-info > 0.4 | 0.94 |
| **ALSPAC-M** | Illumina human660W-quad Beadchip | rs2472297 | Directly genotyped  Call rate : > 95% | 0.1 | rs6968554^*^ | Imputed  Software: MaCH  Reference panel: 1000G, CEU  Quality: 0.996 | 0.51 |
| **HBCS** | Ilumina 610Q | rs2472297 | Imputed  Software: MACH  Reference panel: HapMap 2, CEU  Quality: 0.92 | 0.43 | rs4410790^$^ | Directly genotyped  Call rate: 1.0 | 0.56 |
| **NFBC1966** | Illumina HumanCNV370DUO | rs2472297 | Imputed  Software: IMPUTE  Reference panel: HapMap R22, CEU  Quality: INFO score = 0.90 | 0.64 | rs4410790^$^ | Directly genotyped  Call rate: > > 95% | 0.37 |
| **PIVUS** | Illumina HumanOmniExpress Metabochip | rs2472297 | Directly genotyped Call rate: > 95% | 0.10 | rs6968865 | Imputed  Software: IMPUTE  Reference panel: HapMap R22, CEU | 0.55 |
| **STR** | Illumina iSelect Metabochip | rs2472297 | Directly genotyped  Call rate: > 95% | 0.29 | rs6968554^*^ | Directly genotyped  Call rate: > 95% | 0.65 |
| **TwinGene** | Illumina OmniExpress | rs2472297 | Directly genotyped  Call rate: > 95% | 0.64 | rs6968554^*^ | Directly genotyped  Call rate: > 95% | 0.75 |
| **UK Biobank** | UK Biobank Axiom array (~ 450,000 samples) UK BiLEVE Axiom array (~ 50,000 samples) | rs2472297 | Directly genotyped  Call rate: 99.8% | 0.20 | rs6968554^*^ | Imputed  Software: IMPUTE3 (modified from IMPUTE2 with a greater computational efficiency)  Reference panel: UK10K haplotype and 1000G (Phase 3) | 0.35 |
| **ULSAM** | Illumina Omni 2.5 M Metabochip | rs2472297 | Directly genotyped Call rate: 99.7% | 0.07 | rs6968554^*^ | Directly genotyped  Call rate: 99.8% | 0.68 |
| **YFS** | Illumina custom BeadChip, 670Q | rs2472297 | Directly genotyped  Call rate: > 95% | 0.85 | rs6968865 | Imputed  Software: IMPUTE2  Reference panel: 1000G phase I integrated release version 3 | 0.40 |

^*^SNP proxy for rs6968865 with R^2^ = 1.0; ^$^SNP proxy for rs6968865 with R^2^ = 0.97; HWE: Hardy Weinberg Equilibrium

## Table S2. Coffee intake information in each cohort

| **Study** | **Coffee questionnaire** |
| --- | --- |
| **1958BC** | How often do you drink coffee? (choice response) > 4 times a day  2-4 times a day once a day 3-6 days a week 1 or 2 days a week less than 1 day a week occasionally never |
| **ALSPAC-M** | How many cups of coffee (real, instant or decaffeinated) do you drink? (response in cups per day) |
| **HBCS** | Average coffee intake over the past year  never  1-3 cups/month  1 cup/week  2-4 cups/week  5-6 cups/week  1 cup/day  2-3 cups/day  4-5 cups/day  6+ cups/day |
| **NFBC1966** | How many cups of coffee do you usually drink in a day? filtered coffee: integer response boiled coffee: integer response |
| **PIVUS** | 7-day dietary records: Coffee intake (cups) was recorded 6 times daily (breakfast, lunch, supper, between meals, and in the evening). (integer response) |
| **STR (SATSA + GENDER)** | How many cups of coffee do you drink a day? (integer response) |
| **TwinGene** | How many cups of coffee do you usually drink a day?  (choice response)  never  1 cup/day  2 cups/day  3 cups/day  4 cups/day  5 cups/day  (if more than 5 provide precise amount)  don't know  refuse |
| **UK Biobank** | How many cups of coffee do you drink each day? (Include decaffeinated coffee) |
| **ULSAM** | 7-day dietary records: Coffee intake (cups) was recorded 6 times daily (breakfast, lunch, supper, between meals, and in the evening). |
| **YFS** | Average coffee intake (1 cup) over the past year  (choice response) Never or less frequently 1-3 cups/month 1 cup/week 2-4 cups/week 5-6 cups/week 1 cup/day 2-3 cups/day 4-5 cups/day  6+ cups/day |

Note: Similar questions were used to obtain information on tea consumption.

## Table S3. Cognitive measures for global and memory cognition

| **Study** | **Study design** | **Country** | **Cognitive tests for global cognition** | **Cognitive tests for memory cognition** |
| --- | --- | --- | --- | --- |
| **1958BC** | Perspective birth cohort study | UK | Immediate Word Recall  Delayed Word Recall  Verbal Fluency | Immediate Word Recall  Delayed Word Recall |
| **ALSPAC-M** | Perspective birth cohort | UK | Logic Memory 1 and 2  Backwards Digit Span  Digit Symbol Coding  Spot-The-Word Test  Verbal Fluency Test | Logic Memory 1 and 2 |
| **HBCS** | Perspective birth cohort study | Finland | Immediate Verbal Recall  Delayed Verbal Recall  Verbal Learning  Verbal Fluency | Immediate Verbal Recall  Delayed Verbal Recall  Verbal Learning |
| **NFBC1966** | Perspective birth cohort study | Finland | Paired Associates Learning Test  (Visual Memory and New Learning) | Paired Associates Learning Test  (Visual Memory and New Learning) |
| **PIVUS** | Cohort study | Sweden | Mini-Mental State Examination  7-Minute Test (Benton Temporal Orientation, Clock Drawing, and Verbal Fluency)  Trail Making Test part A Trail Making Test part B | Enhanced Cued Recall (only time was used, without reminder) |
| **STR (SATSA + GENDER)** | Cohort study | Sweden | The first component of cognitive measurement scale | Immediate Word Recall (SATSA)  Delayed Word Recall (SATSA)  Thurstone's Memory Test (GENDER) |
| **TwinGene** | Cohort study | Sweden | A cognitive screening score constructed from Immediate Word Recall, Delayed Word Recall, Similarities, Serial Threes, etc | Immediate Word Recall  Delayed Word Recall |
| **UK Biobank** | Population-based cohort study | UK | Pairs Matching Test Reaction Time Test | Pairs Matching Test |
| **ULSAM** | Cohort study | Sweden | Mini-Mental State Examination Trail Making Test |  |
| **YFS** | Population-based cohort study | Finland | Paired Associates Learning Test  Spatial Working Memory Test  Reaction Time Test  Rapid Visual Information Test | Paired Associates Learning Test |

## Table S4. Depression, education and study-specific covariates in each cohort

| **Study** | **Depression** | **Education** | **Study-specific covariates** |
| --- | --- | --- | --- |
| **1958BC** | Assessed using the Clinical Interview Schedule-Revised at 45 years ^31,32^ | Highest qualification obtained by 42 years, or by 33 years if data was missing.  Five categories, including:  1) None  2) Some qualifications  3) O-level / equivalent  4) A-level / equivalent  5) Degree | None |
| **ALSPAC-M** | Not available | Three categories:  1) Certificate of secondary education/vocational qualifications  2) O-level  3) A-level / degree | None |
| **HBCS** | Assessed using Centre for Epidemiologic Studies Depression Scale with a cut-off of <16. | Three categories:  1) Folk school / elementary / middle school  2) Learning profession, elementary school or similar  3) Lower or higher university degree | None |
| **NFBC1966** | Taken from the question “Have you ever had any of the following symptoms, sicknesses or injuries verified or treated by a doctor: depression?” | Three categories:  1) No occupational education / vocational training course  2) Vocational school / post-secondary education  3) Polytechnic education / university degree | None |
| **PIVUS** | Not available | Three categories:  1) Primary school  2) Secondary school  3) University | None |
| **STR**  **(SATSA + GENDER)** | Review of medical record in the National Patient Register  Defined using Centre for Epidemiologic Studies Depression Scale | Three categories:  1) Elementary or middle school  2) High school or equivalent  3) College or higher | Study indicator (SATAS or GENDER) |
| **TwinGene** | Defined using Centre for Epidemiologic Studies Depression Scale, usage of any antidepressant, or mental disorder screening items | Three categories:  1) Elementary or middle school  2) High school or equivalent  3) College or higher | None |
| **UK Biobank** | Derived from the question “In the last 2 weeks, how often have you felt down, depressed or hopeless?  1) Not at all  2) Several days  3) More than half the days  4) Nearly every day  5) Do not know  6) Prefer not to answer  Depression = several days / more than half the days /nearly every day  No depression = not at all / do not know  Missing = prefer not to answer | Three categories:  1) None  2) National vocational qualification / certification of secondary education / O-levels / A-levels  3) Degree / professional | SNP array indicator (UK Biobank Axiom array or UK BiLEVE Axiom array |
| **ULSAM** | Derived from the questionnaire on living conditions including the following questions: Are you happy with your day-to-day existence?  1) Yes  2) Yes, mostly  3) Yes, sometimes  4) No, almost never  Depression = yes / yes, mostly / yes, sometimes  No depression = no, almost never | Three categories:  1) Years of education ≤ 7 yrs  2) Years of education = 8 -10 yrs  3) Years of education ≥13 yrs | None |
| **YFS** | Taken from self-reported data on depression diagnoses in 2007 | Three categories (year 2007)  1) Vocational school / occupational/vocational college / basic education  2) University of applied sciences / university studies (no final degree) / lower university degree (Bachelor’s degree)  3) Higher university degree (Masters) / licentiate degree / doctoral degree | None |

# Supplementary Results

Eight genome-wide significant loci related to habitual coffee intake

(*AHR*, *CYP1A1/2*, *POR*, *EFCAB5*, *GCKR*, *ABCG2*, *MLXIPL*, and *BDNF*)

Pleiotropic

Not pleiotropic

No

Yes

Caffeine metabolism

*GCKR*, *ABCG2*, *MLXIPL* and *BDNF*

*POR* and *EFCAB5*

*AHR* and *CYP1A1/2*

Usage of variants as instruments for coffee intake in our study

**Primary MR analyses in all 10 cohorts:**

- Association of genetic score (=*AHR* + *CYP1A1/2*) with cognitive outcomes

**Secondary MR analyses in the UK Biobank:**

1. Association of *POR* and *EFCAB5* with cognitive outcomes
2. MR-Egger regression using *AHR*, *CYP1A1/2*, *POR*, *EFCAB5*, *GCKR*, *ABCG2*, *MLXIPL*, and *BDNF*

## Figure S1. Usage of genome-wide significant coffee variants as genetic instruments for habitual coffee intake.

**a**

**b**

## Figure S2. Association of different genetic instruments with global (a) and memory (b) cognitive scores, among coffee drinkers in the UK Biobank.

Error bars are the 95% confidence intervals**.**

## Figure S3. Association of different genetic instruments with prospective memory, among coffee drinkers in the UK Biobank.

Error bars are the 95% confidence intervals**.**

**a**

**b**

## Figure S4. Association of *AHR*, *CYP1A1/2* and genetic score with habitual tea (a) and caffeine (b) intake.

Error bars are the 95% confidence intervals.

## Figure S5. Association of *AHR*, *CYP1A1/2* and genetic score with global and memory cognitive scores, among coffee, tea and caffeine consumers.

Error bars are the 95% confidence intervals.

**a**

**b**

## Figure S6. Association of genetic score with global (a) and memory (b) cognitive scores from the crude model, among coffee drinkers in individual cohort.

Error bars are the 95% confidence intervals.

**a**

**b**

## Figure S7. Association of genetic score with global (a) and memory (b) cognitive scores from the adjusted model, among coffee drinkers in individual cohort.

Error bars are the 95% confidence intervals.

## Table S5. Association of *AHR*, *CYP1A1/2*, and genetic score with habitual coffee intake in individual cohort

| **Coffee variants** | **Study** | **N** | **β^*^ (95% C.I.)** | **P** |
| --- | --- | --- | --- | --- |
| *AHR* | 1958BC | 2,861 | 0.09 (0.04 , 0.15) | 1.3x10^-3^ |
|  | ALSPAC Mothers | 1,333 | 0.13 (-0.01 , 0.27) | 7.3x10^-2^ |
|  | UK Biobank | 290,576 | 0.11 (0.1 , 0.12) | 4.5x10^-308^ |
|  | HBCS | 760 | 0.15 (0.01 , 0.28) | 3.0x10^-2^ |
|  | NFBC1966 | 1,697 | 0.41 (0.22 , 0.61) | 4.3x10^-5^ |
|  | YFS | 1,754 | 0.25 (0.08 , 0.41) | 3.3x10^-3^ |
|  | PIVUS | 719 | 0.05 (-0.12 , 0.21) | 6.0x10^-1^ |
|  | ULSAM | 1,020 | 0.19 (0.05 , 0.32) | 7.3x10^-3^ |
|  | STR | 952 | 0.27 (0.08 , 0.45) | 4.8x10^-3^ |
|  | TwinGene | 2,215 | 0.17 (0.08 , 0.26) | 4.2x10^-4^ |
| *CYP1A1/2* | 1958BC | 2,861 | 0.11 (0.05 , 0.17) | 7.1x10^-4^ |
|  | ALSPAC Mothers | 1,333 | 0.14 (-0.02 , 0.29) | 8.0x10^-2^ |
|  | UK Biobank | 290,576 | 0.14 (0.13 , 0.15) | 4.5x10^-308^ |
|  | HBCS | 760 | 0.06 (-0.11 , 0.22) | 5.0x10^-1^ |
|  | NFBC1966 | 1,697 | 0.37 (0.15 , 0.58) | 8.1x10^-4^ |
|  | YFS | 1,754 | 0.27 (0.09 , 0.44) | 3.1x10^-3^ |
|  | PIVUS | 719 | 0.06 (-0.12 , 0.25) | 5.0x10^-1^ |
|  | ULSAM | 1,020 | 0.18 (0.03 , 0.33) | 1.7x10^-2^ |
|  | STR | 952 | 0.28 (0.06 , 0.49) | 1.1x10^-2^ |
|  | TwinGene | 2,215 | 0.17 (0.07 , 0.26) | 1.1x10^-3^ |
| Genetic score | 1958BC | 2,861 | 0.1 (0.06 , 0.14) | 4.2x10^-6^ |
|  | ALSPAC Mothers | 1,333 | 0.13 (0.03 , 0.24) | 1.3x10^-2^ |
|  | UK Biobank | 290,576 | 0.12 (0.12 , 0.13) | 4.5x10^-308^ |
|  | HBCS | 760 | 0.12 (0.01 , 0.23) | 3.0x10^-2^ |
|  | NFBC1966 | 1,697 | 0.39 (0.24 , 0.53) | 1.7x10^-7^ |
|  | YFS | 1,754 | 0.25 (0.13 , 0.37) | 3.4x10^-5^ |
|  | PIVUS | 719 | 0.05 (-0.07 , 0.18) | 4.0x10^-1^ |
|  | ULSAM | 1,020 | 0.19 (0.09 , 0.29) | 2.6x10^-4^ |
|  | STR | 952 | 0.28 (0.14 , 0.42) | 1.1x10^-4^ |
|  | TwinGene | 2,215 | 0.17 (0.11 , 0.24) | 8.3x10^-7^ |

^*^Δ cups/day per intake-increase allele

## Table S6. Association of *AHR*, *CYP1A1/2* and genetic score with confounders in the UK Biobank study

| **Coffee variants** | **N** | **Males**  **(%)** | **Age, years**  **Mean (SD)** | **Education** | | **Smokers**  **(%)** | **Depression**  **(%)** |
| --- | --- | --- | --- | --- | --- | --- | --- |
|  |  |  |  | **Medium^*^**  **(%)** | **High^**^**  **(%)** |  |  |
| *AHR* |  |  |  |  |  |  |  |
| AA | 53,336 | 45.69 | 56.9 (8.0) | 35.5 | 46.6 | 10.3 | 21.6 |
| AG | 187,511 | 45.98 | 56.9 (8.0) | 36.0 | 46.3 | 10.1 | 22.0 |
| GG | 163,773 | 46.07 | 57.0 (8.0) | 36.2 | 45.9 | 10.2 | 22.1 |
|  |  | *P=0.32* | *P=0.43* |  | *P=0.005* | *P=0.08* | *P=0.06* |
| *CYP1A1/2* |  |  |  |  |  |  |  |
| CC | 217,620 | 45.9 | 56.9 (8.0) | 36.0 | 46.2 | 10.1 | 22.0 |
| CT | 158,017 | 46.0 | 56.9 (8.0) | 36.0 | 46.2 | 10.1 | 21.9 |
| TT | 28,983 | 46.1 | 56.9 (7.9) | 36.2 | 45.8 | 10.3 | 22.4 |
|  |  | *P=0.81* | *P=0.44* |  | *P=0.74* | *P=0.91* | *P=0.16* |
| Genetic Score |  |  |  |  |  |  |  |
| 0 | 28,849 | 45.62 | 56.9 (8.0) | 35.2 | 46.7 | 10.3 | 21.8 |
| 1 | 121,723 | 45.92 | 56.9 (8.0) | 36.0 | 46.5 | 10.1 | 21.9 |
| 2 | 164,681 | 45.98 | 56.9 (8.0) | 36.1 | 46.0 | 10.2 | 22.0 |
| 3 | 77,513 | 46.19 | 56.9 (8.0) | 36.1 | 46.0 | 10.2 | 22.2 |
| 4 | 11,854 | 45.92 | 56.8 (8.0) | 36.4 | 45.7 | 9.8 | 22.4 |
|  |  | *P=0.54* | *P=0.95* |  | *P=0.016* | *P=0.046* | *P=0.32* |

SD: standard deviation; ^*^Medium = national vocational qualification / certification of secondary education / O-levels / A-levels ; ^**^High = degree / professional

Note: Bonferroni corrected type 1 error = 0.05/15 = 0.003, meaning P values less than 0.003 are considered statistically significant.

# ACKNOWLEDGEMENT

This study was funded by J.J. Mason and H.S. Williams Memorial Foundation grant CT23158. **1958BC:** This work made use of data and samples generated by the 1958 Birth Cohort (NCDS), which is managed by the Centre for Longitudinal Studies at the UCL Institute of Education, funded by the Economic and Social Research Council (grant number ES/M001660/1). Access to these resources was enabled via the 58READIE Project funded by Wellcome Trust and Medical Research Council (grant numbers WT095219MA and G1001799). A full list of the financial, institutional and personal contributions to the development of the 1958 Birth Cohort Biomedical resource is available at [http://www2.le.ac.uk/projects/birthcohort/1958bc/about/ contributors-funders](http://www2.le.ac.uk/projects/birthcohort/1958bc/about/%20contributors-funders). The Medical Research Council funded the 2002–2004 clinical follow-up of the 1958 birth cohort (grant G0000934). Genotyping was undertaken as part of the Wellcome Trust Case-Control Consortium (WTCCC) under Wellcome Trust award 076113, and a full list of the investigators who contributed to the generation of the data is available at [www.wtccc.org.uk](http://www.wtccc.org.uk/). This research used resources provided by the Type 1 Diabetes Genetics Consortium, a collaborative clinical study sponsored by the National Institute of Diabetes and Digestive and Kidney Diseases (NIDDK), National Institute of Allergy and Infectious diseases, National Human Genome Research Institute, National Institute of Child Health and Human Development, and Juvenile Diabetes Research Foundation International (JDRF) and supported by U01DK062418. The research was supported by the National Institute for Health Research Biomedical Research Centre at Great Ormond Street Hospital for Children NHS Foundation Trust and University College London. DJL acknowledges funding from the Mary Kinross Charitable Trust and the Halpin Trust. **ALSPAC-M:** We are extremely grateful to all the families who took part in this study, the midwives for their help in recruiting them, and the whole ALSPAC team, which includes interviewers, computer and laboratory technicians, clerical workers, research scientists, volunteers, managers, receptionists and nurses. The UK Medical Research Council and the Wellcome Trust (Grant ref: 102215/2/13/2) and the University of Bristol provide core support for ALSPAC. This publication is the work of the authors and AET will serve as guarantors for the contents of this paper. AET and MRM are members of the UK Centre for Tobacco Control Studies, a UKCRC Public Health Research: Centre of Excellence. Funding from British Heart Foundation, Cancer Research UK, Economic and Social Research Council, Medical Research Council, and the National Institute for Health Research, under the auspices of the UK Clinical Research Collaboration, is gratefully acknowledged. This work was supported by the Medical Research Council (MC_UU_12013/6). **NFBC1966:** The NFBC resource has been supported by grants from the Academy of Finland (project grants 104781, 120315, 129269, 1114194, 24300796, Center of Excellence in Complex Disease Genetics and SALVE), University Hospital Oulu, Biocenter, University of Oulu, Finland (75617), NHLBI grant 5R01HL087679-02 (1RL1MH083268-01), NIH/NIMH (5R01MH63706:02), ENGAGE project and grant agreement HEALTH-F4-2007-201413, EU FP7 EurHEALTHAgeing -277849, the Medical Research Council, UK (G0500539, G0600705, G1002319, PrevMetSyn/SALVE) and the MRC, Centenary Early Career Award. H2020 DynaHEALTH (European Union’s Horizon 2020 research and innovation programme under grant agreement No 633595); Exposomic, Genomic and Epigenomic Approach to Prediction of Metabolic and Cardiorespiratory function and Ill-Health (EGEA), Academy of Finland, Grant No 285547; ALEC Study (funded by the European Union's Horizon 2020 Research and Innovation programme under grant agreement No. 633212); H2020 / Marie Skłodowska-Curie Actions, CAPICE (Marie Curie Grant agreement Number 721567); National Public Health Institute, Biomedicum Helsinki, Finland. We thank the late Professor Paula Rantakallio (launch of NFBCs), and Ms Outi Tornwall and Ms Minttu Jussila (DNA biobanking). The authors would like to acknowledge the contribution of the late Academian of Science Leena Peltonen. **HBCS:** We thank all study participants as well as everybody involved in the Helsinki Birth Cohort Study. Helsinki Birth Cohort Study has been supported by grants from the Academy of Finland, the Finnish Diabetes Research Society, Folkhälsan Research Foundation, Novo Nordisk Foundation, Finska Läkaresällskapet, Juho Vainio Foundation, Signe and Ane Gyllenberg Foundation, University of Helsinki, Ministry of Education, Ahokas Foundation, Emil Aaltonen Foundation. **STR:** SATSA is supported by National Institute of Aging (AG04563, AG10175), The MacArthur Foundation Research Network on Successful Aging, Swedish Research Council (825-2007-7460, 825-2009-6141, 825-3011-6182,521-2013-8689, 2015-03255), and the Swedish Council for Working Life and Social Research (FAS/FORTE) (97:0147:1B, 2009-0795,2013-2292). GENDER is supported by the MacArthur Foundation Research Network on Successful Aging, the Axel and Margaret Ax:son Johnsons Foundation, the Swedish Council for Social Research and the Swedish Foundation for Health Care Sciences and Allergy Research. **TwinGene:** TwinGene was supported by the Swedish Research Council (M-2005-1112 and 2009-2298), GenomEUtwin (EU/QLRT-2001-01254; QLG2-CT-2002-01254), National Institutes of Health (grant DK U01-066134), Swedish Foundation for Strategic Research (SSF; ICA08-0047). **UK Biobank:** This research has been conducted using the UK Biobank Resource (applications 9142 and 10171). We would like to thank Dr Thomas Littlejohns for his advice on the optimal use of the UK Biobank data. **YFS:** The Young Finns Study was supported by the Academy of Finland: grants 286284, 134309 (Eye), 126925, 121584, 124282, 129378 (Salve), 117787 (Gendi), and 41071 (Skidi); the Social Insurance Institution of Finland; Competitive State Research Financing of the Expert Responsibility area of Kuopio, Tampere and Turku University Hospitals (grant X51001); Juho Vainio Foundation; Paavo Nurmi Foundation; Finnish Foundation for Cardiovascular Research ; Finnish Cultural Foundation; Tampere Tuberculosis Foundation; Emil Aaltonen Foundation; Yrjö Jahnsson Foundation; Signe and Ane Gyllenberg Foundation; and Diabetes Research Foundation of Finnish Diabetes Association.

# REFERENCES

1 Power, C. & Elliott, J. Cohort profile: 1958 British birth cohort (National Child Development Study). *Int. J. Epidemiol.* **35**, 34-41 (2006).

2 Genome-wide association study of 14,000 cases of seven common diseases and 3,000 shared controls. *Nature* **447**, 661-678 (2007).

3 Barrett, J. C. *et al.* Genome-wide association study and meta-analysis find that over 40 loci affect risk of type 1 diabetes. *Nature genetics* **41**, 703-707 (2009).

4 Ofstedal, M. B., Fisher, G. G. & Herzog, A. R. Documentation of Cogntive Functioning Measures in the Health and Retirement Study. (2005).

5 Roth, M. *CAMDEX-R: the Cambridge examination for mental disorders of the elderly*. (Cambridge University Press, 1998).

6 Richards, M., Kuh, D., Hardy, R. & Wadsworth, M. Lifetime cognitive function and timing of the natural menopause. *Neurology* **53**, 308 (1999).

7 Boyd, A. *et al.* Cohort Profile: the 'children of the 90s'--the index offspring of the Avon Longitudinal Study of Parents and Children. *International journal of epidemiology* **42**, 111-127 (2013).

8 Fraser, A. *et al.* Cohort Profile: the Avon Longitudinal Study of Parents and Children: ALSPAC mothers cohort. *International journal of epidemiology* **42**, 97-110 (2013).

9 Purcell, S. *et al.* PLINK: a tool set for whole-genome association and population-based linkage analyses. *American journal of human genetics* **81**, 559-575 (2007).

10 Weschsler, D. *Wechsler Memory Scale - Third Edition (UK): Administration and scoring manual*. (The Psychological Corporation, 1998).

11 Wechsler, D. *WAIS-IIIUK administration and scoring manual*. (The Psychological Corporation, 1998).

12 Baddeley, A., Emslie, H. & Nimmo-Smith, I. The Spot-the-Word test: a robust estimate of verbal intelligence based on lexical decision. *The British journal of clinical psychology* **32 ( Pt 1)**, 55-65 (1993).

13 Lezak, M. D., Howieson, D. B., & Loring, D. W. *Neuropsychological Assessment*. 4th edition edn, (Oxford University Press, 2004).

14 Barker, D. J., Osmond, C., Forsén, T. J., Kajantie, E. & Eriksson, J. G. Trajectories of growth among children who have coronary events as adults. *N. Engl. J. Med.* **353**, 1802-1809 (2005).

15 Rantakallio, P. Groups at risk in low birth weight infants and perinatal mortality. *Acta paediatrica Scandinavica* **193**, Suppl 193:191+ (1969).

16 Lind, L., Fors, N., Hall, J., Marttala, K. & Stenborg, A. A comparison of three different methods to evaluate endothelium-dependent vasodilation in the elderly: the Prospective Investigation of the Vasculature in Uppsala Seniors (PIVUS) study. *Arteriosclerosis, thrombosis, and vascular biology* **25**, 2368-2375 (2005).

17 Folstein MF, F. S., McHugh PR. *Mini-Mental State: a practical method for grading the cognitive state of patients for the clinician*. (Pergamon Press, 1975).

18 Solomon, P. R. *et al.* A 7 minute neurocognitive screening battery highly sensitive to Alzheimer's disease. *Archives of neurology* **55**, 349-355 (1998).

19 Lichtenstein, P. *et al.* The Swedish Twin Registry: a unique resource for clinical, epidemiological and genetic studies. *Journal of internal medicine* **252**, 184-205 (2002).

20 Gold, C. H., Malmberg, B., McClearn, G. E., Pedersen, N. L. & Berg, S. Gender and health: a study of older unlike-sex twins. *The journals of gerontology. Series B, Psychological sciences and social sciences* **57**, S168-176 (2002).

21 Pedersen, N. L. *et al.* The Swedish Adoption Twin Study of Aging: an update. *Acta geneticae medicae et gemellologiae* **40**, 7-20 (1991).

22 Magnusson, P. K. *et al.* The Swedish Twin Registry: establishment of a biobank and other recent developments. *Twin research and human genetics : the official journal of the International Society for Twin Studies* **16**, 317-329 (2013).

23 Sudlow, C. *et al.* UK biobank: an open access resource for identifying the causes of a wide range of complex diseases of middle and old age. *PLoS medicine* **12**, e1001779 (2015).

24 Mansournia, M. A. & Altman, D. G. Inverse probability weighting. *BMJ (Clinical research ed.)* **352**, i189 (2016).

25 Manichaikul, A. *et al.* Robust relationship inference in genome-wide association studies. *Bioinformatics (Oxford, England)* **26**, 2867-2873 (2010).

26 Cullen, B. *et al.* Cognitive function and lifetime features of depression and bipolar disorder in a large population sample: Cross-sectional study of 143,828 UK Biobank participants. *European psychiatry : the journal of the Association of European Psychiatrists* **30**, 950-958 (2015).

27 Lyall, D. M. *et al.* Cognitive Test Scores in UK Biobank: Data Reduction in 480,416 Participants and Longitudinal Stability in 20,346 Participants. *PloS one* **11**, e0154222 (2016).

28 Byberg, L. *et al.* Plasminogen activator inhibitor-1 activity is independently related to both insulin sensitivity and serum triglycerides in 70-year-old men. *Arteriosclerosis, thrombosis, and vascular biology* **18**, 258-264 (1998).

29 Universitet, U. *ULSAM-70*, <[http://www2.pubcare.uu.se/ULSAM/invest/70yrs/meth70.htm - 15](http://www2.pubcare.uu.se/ULSAM/invest/70yrs/meth70.htm#15).> (

30 Raitakari, O. T. *et al.* Cohort profile: the cardiovascular risk in Young Finns Study. *International journal of epidemiology* **37**, 1220-1226 (2008).

31 Goldberg, D. P., Cooper, B., Eastwood, M. R., Kedward, H. B. & Shepherd, M. A standardized psychiatric interview for use in community surveys. *Br. J. Prev. Soc. Med.* **24**, 18-23 (1970).

32 Lewis, G., Pelosi, A. J., Araya, R. & Dunn, G. Measuring psychiatric disorder in the community: a standardized assessment for use by lay interviewers. *Psychol. Med.* **22**, 465-486 (1992).
